# Polyamines, Alu elements and nucleolar disruption in Alzheimer's disease

**DOI:** 10.1002/alz70855_098824

**Published:** 2025-12-23

**Authors:** Wesley H Brooks

**Affiliations:** ^1^ University of South Florida, Safety Harbor, FL, USA

## Abstract

**Background:**

The “X chromosome‐nucleolus nexus” hypothesis for lupus, describing mutual disruption of the inactive X and nucleoli, can assist in understanding Alzheimer's etiology. Since 49% of cellular pathways are affected in Alzheimer's, a systems approach is required, looking at multiple pathways and shared reactants like S‐adenosylmethionine (SAM) and acetyl‐CoA. Here, a new hypothesis is proposed for Alzheimer's describing mutual disruption of nucleoli and Alzheimer's related genes in peri‐nucleolar chromatin.

**Method:**

Key word searches (e.g., PubMed) retrieved relevant publications. Chromosome locations of Alzheimer's related genes and Alu element clusters were determined.

**Result:**

The hypothesis “polyamine dysregulation and nucleolar disruption” emerged in which cellular stress can induce increased polyamine metabolism that wastes SAM, used in polyamine synthesis, and acetyl‐CoA, used in polyamine recycling. Low SAM induces p38 kinase phosphorylation of Tau. Low acetyl‐CoA leads to low acetylcholine seen in Alzheimer's. Polyamine changes drive nucleolar dynamics which disrupts peri‐nucleolar chromatin. This can open clusters of Alu elements which can be expressed in abundance by RNA polymerase III. Alu RNA transcripts can bind nucleolin in competition with structural RNAs that normally bind nucleolin to stabilize the nucleolar shell. As the nucleolus loses its integrity, it becomes very inefficient, even fragmenting. In lupus this can release autoantigens, many of which contain nucleolar components. Alu elements comprise 11% of the genome but Alu clusters exist, for example, in chromosome 19 with the ApoE4 allele, Alu elements comprise 25.8%. Nucleolar disruption can disrupt epigenetic control of Alzheimer's related genes in peri‐nucleolar chromatin, such as PSEN1 on chromosome 14 and APP on chromosome 21. With the appearance of hyperphosphorylated Tau, there can be aggregation by increased polyamines.

**Conclusion:**

The “polyamine dysregulation and nucleolar disruption” hypothesis describes how stress can lead to extraordinary nucleolar dynamics, disrupting epigenetic control of Alzheimer's related genes. In addition, Alu clusters reside in peri‐nucleolar chromatin with the possibility that significant Alu expression could further disrupt the nucleoli. Also, there are sequestered polyamine metabolism alleles located in the inactive X that could become active. Wasteful polyamine metabolism reduces SAM and acetyl‐CoA leading to Tau hyperphosphorylation by p38 kinase and low acetylcholine seen in Alzheimer's.

